# Pharmacological alternatives to oxytetracycline as potential treatment of flexural limb deformities in foals: a preliminary in vitro cell viability and proliferation study

**DOI:** 10.1038/s41598-025-00311-z

**Published:** 2025-05-06

**Authors:** Emmanuel Mathieu Cardinaux, Hilke Oltmanns, Andreas Beineke, Jessica Meißner, Florian Geburek

**Affiliations:** 1 Clinic for Horses, University of Veterinary Medicine Hannover, Foundation, Hannover, Germany; 2 Department of Pharmacology, Toxicology and Pharmacy, University of Veterinary Medicine Hannover, Foundation, Hannover, Germany; 3 Department of Pathology, University of Veterinary Medicine Hannover, Foundation, Hannover, Germany

**Keywords:** Flexural limb deformities, Foals, Oxytetracycline, Matrix-metalloproteinase-inhibitors, Preclinical research, Drug discovery, Toxicology

## Abstract

Flexural limb deformities are a widespread condition in foals. Oxytetracycline is a common conservative treatment option with relaxing effects on the muscle-tendon-unit, potentially mediated through a matrix-metalloproteinase (MMP)-inhibitor mechanism. Its high therapeutic dose for this indication, potential negative side effects, and the guidelines for prudent use of antimicrobials make investigating alternatives desirable. In this study, the influence of substances with potentially similar mechanisms of action, however without antimicrobial properties, on viability and proliferation of juvenile myofibroblasts was assessed in vitro. Myofibroblasts from forelimb superficial digital flexor tendons and accessory ligaments of the deep digital flexor tendon from 6 foals, euthanized for reasons unrelated to this study, were cultured and characterized. The myofibroblasts were incubated with oxytetracycline, the MMP-inhibitors incyclinide, ilomastat, aprotinin, pentoxifylline, the lathyrogenic agent β-aminopropionitrile fumarate and Dulbecco’s modified eagle medium as control. Colorimetric cell viability (MTS) and crystal violet assays assessed their viability and proliferation capacities. The morphology and immunohistochemistry profile of the cultured cells was consistent with tendon and ligament myofibroblasts. All test substances were biocompatible, shown by the absence of significant differences with cells incubated with medium, demonstrating the absence of cytotoxic or anti-proliferative effect on juvenile myofibroblasts in the tested concentrations in this preliminary study.

## Introduction

Flexural limb deformities are a widespread orthopaedic condition in foals^1^. The metacarpophalangeal and distal interphalangeal joints are most frequently affected, and the pathology can be congenital or acquired. Before surgical therapy is required, conservative measures can be taken successively and synergistically, including pain relief, controlled exercise, immobilisation of the affected limb, corrective hoof trimming, application of acrylic/glue-on shoes, and intravenous administration of oxytetracycline (OTC)^1–4^.

The relaxing effect of OTC on the muscle-tendon-unit of foals affected by flexural limb deformities was discovered by accident in the 1970s and described in the 1980s. Lokai and Meyer (1985) reported the successful treatment of 29 foals affected by congenital flexural deformities using an intravenous injection of 3 g of oxytetracycline^4^. These preliminary observations could be confirmed in vivo radiographically and kinematically in subsequent studies^5,6^. Moreover, another study demonstrated *post-mortem* an increased elasticity of adult deep digital flexor muscle-tendon-units following in vivo treatment with OTC compared to controls, while no effect on the crosslink deoxypyridinoline content in the urine of OTC-treated adult horses could be found^7^.

However, the mechanism of action of OTC on the muscle-tendon-unit has not been fully clarified. Arnoczky et al. (2004) showed that OTC induced a dose-dependent reduction in matrix-metalloproteinase (MMP)-1 mRNA expression by myofibroblasts of juvenile muscle-tendon-units^8^. This inhibition of MMPs affects the remodelling and tractional structuring of collagen by myofibroblasts, the predominant cells in juvenile equine tendons^9^, and make the tendons more vulnerable to creep^8^. Other mechanisms such as the inhibition of calcium mediated muscle contraction through a calcium chelation^10^, a neuromuscular blockage^11^, or an interaction with collagen cross-linking, have been suggested^12,13^.

Nevertheless, the use of OTC for this indication is a point of debate. Following the guidelines for prudent use of antimicrobials and with the aim to reduce their quantity, the administration of an antimicrobial for another purpose is justifiable only under specific conditions^14^. Moreover, the therapeutic dose of OTC for flexural limb deformities is higher than the antimicrobial dose (40–70 mg/kg bwt. vs. 6.6 mg/kg bwt.), increasing the risk of potentially life-threatening side effects in foals such as acute renal failure^15,16^.

Consequently, investigating alternative substances with potentially similar mechanisms of action on flexural deformities, however without antimicrobial properties, is of ethical, clinical, and economic importance. Different substances used in various fields present MMP-inhibitor properties and could be interesting candidates. Among them the chemically modified tetracyclines (CMTs) incyclinide (CMT-3, COL-3)^17,18^, the hydroxamate Ilomastat^19^, the protease inhibitor aprotinin^20^, or the xanthine derivative pentoxifylline, used in equine medicine for the treatment of laminitis, endometritis, placentitis or vasculitis due to its vasoactive properties^21^, all show MMP-inhibitor properties and have been studied in the fields of human oncology, cardiology, dentistry, ophthalmology or wound healing. The mechanism of action of lathyrogens differs from MMP-inhibitors as they cause decreased mechanical strength and increased collagen solubility by interacting with collagen cross-linking. They could, however, show similarities to oxytetracycline as suggested by Wintz et al. (2012) in an investigation of the effects of oxytetracycline on the viscoelastic properties of rat tail tendons^13^. Beta-aminopropionitrile fumarate (BAPN), the toxic constituent of the sweat pea plant (Lathyrus odoratus), is a representant of this family and has been used as intralesional treatment of equine tendinopathy^13,22^.

In order to investigate the potential of such substances as alternatives to OTC for the treatment of flexural limb deformities in foals and before assessing their relaxing influence on the muscle-tendon-unit in foals, it is essential to assess their biocompatibility with myofibroblasts of juvenile equine tendons and ligaments. Consequently, the goal of this study was twofold. The first goal was to culture and characterize through immunohistochemistry myofibroblasts from the superficial digital flexor tendon (SDFT) and the accessory ligament of the deep digital flexor tendon (ALDDFT), or distal check ligament, of foals. The second goal was to assess through in vitro MTS and crystal violet assays the influence of the aforementioned substances on the viability and proliferation of the cultured juvenile myofibroblasts.

## Materials and methods

### Cell culture

The SDFT and the respective ALDDFT of the forelimbs of 6 warmblood foals, aged between 2 and 85 days (median 8 days) were aseptically harvested within 6 h of death and stored in Dulbecco’s modified eagle medium (DMEM) (Carl Roth GmbH, Karlsruhe, Germany) with 1% penicillin/streptomycin (10.000 I.U./mL / 10.000 µg/mL, Bio&Sell GmbH, Feucht, Germany) for transport. The foals were euthanized at the Clinic for Horses of the University of Veterinary Medicine Hannover and at the stud farm Gestüt Lewitz (Neustadt-Glewe, Germany) in a clinical setting for reasons unrelated to this study including pneumonia, neonatal maladjustment syndrome, ruptured bladder, gastro-intestinal anomalies and *rhodococcus equi* infection. After a sedation with xylazine (0.85 mg/kg bwt. i.v.; CP-Pharma Handelsgesellschaft mbH, Burgdorf, Germany) and butorphanol (0.02 mg/kg bwt. i.v; CP-Pharma Handelsgesellschaft mbH, Burgdorf, Germany), the foals were induced into general anaesthesia using ketamine (2.5 mg/kg bwt. i.v.; Vetoquinol GmbH, Ismaning, Germany) and diazepam (0.05 mg/kg bwt. i.v.; TMV Tiergesundheit GmbH, Berlin, Germany) before being euthanized using pentobarbital (100 mg/kg i.v.; CP-Pharma Handelsgesellschaft mbH, Burgdorf, Germany). The experiments were approved as an in vitro study without the use of experimental animals by the animal welfare officer of the University of Veterinary Medicine Hannover and all methods were performed in accordance with the relevant guidelines and regulations.

Myofibroblasts were isolated from these structures and cultured as previously described with some modifications^23^. Briefly, the tendons were washed in phosphate-buffered saline (PBS) and cut into 0.25 cm^3^ pieces with a scalpel blade. The tendon pieces were then incubated with 3 mg/mL collagenase A (Roche diagnostics GmbH, Mannheim, Germany) in DMEM with 1% penicillin/streptomycin at 37° C with 5% CO^2^ for 16–20 h. Subsequently, the cell suspension was filtered through 70 μm and 40 μm nylon mesh filters and centrifuged at 1’000xg for 5 min. The supernatant was discarded, and the cell pellet was resuspended in DMEM with 1% penicillin/streptomycin and 10% fetal bovine serum (Bio&Sell GmbH, Feucht, Germany). The cells were cultured in 25 cm^2^ tissue culture flasks at 37° with 5% CO_2_ as monolayer at 1 million cells/flask.

### Cell imaging

The myofibroblasts as monolayer in culture flasks were imaged by phase-contrast microscopy, and pictures were obtained using a Zeiss Axiocam 105 Color digital camera (Carl Zeiss AG, Oberkochen, Germany) mounted on a Zeiss Axio Vert.A1 inverted phase-contrast microscope (Carl Zeiss AG, Oberkochen, Germany).

### Immunohistochemistry

Superficial digital flexor tendon myofibroblasts of 2 individuals and ALDDFT myofibroblasts of 3 individuals were expanded to passage 1 and immunohistochemistry for the detection of collagen type I, collagen type III and alpha-smooth muscle actin (α-SMA) was performed.

Therefore, the cells were pelleted, fixed in formalin, and embedded in paraffin. The immunohistochemistry staining was performed using the avidin-biotin complex method as described previously^24^. Briefly ROTICLEAR^®^ (Carl Roth, Karlsruhe, Germany) was used to deparaffinize formalin-fixed and paraffin-embedded slides. The slides were then rehydrated using a succession of graded alcohols for 5 min each. Incubation with H_2_O_2_ (0.5%) in 85% ethanol for 30 min was performed in order to suppress endogenous peroxidase activity. For collagen type I only this was followed by antigen retrieval in citrate buffer (pH 6.0) for 20 min in a microwave (800 W). The slides were incubated with goat normal serum at room temperature for 20 min to block unspecific reactions with the secondary antibody. The slides were then incubated with the primary antibodies (collagen I Rockland 1:200, collagen III Acris 1:100, SMA Dako 1:200) diluted in PBS with 1% bovine serum albumin (BSA, Carl Roth) at 4*°* C for 18 h followed with polyclonal biotinylated antibodies diluted 1:200 in PBS (goat anti-mouse, Vector Laboratories, Burlingame, CA, USA, BA-9200; goat anti-rabbit, Vector Laboratories, BA-1000) at room temperature for 45 min. Finally, they were treated with the avidin-biotin-peroxidase complex (Vectastain Elite ABC Kit, Vector Laboratories) at room temperature for 20 min.

### Pharmacological compounds

Oxytetracycline (Sigma Aldrich Steinheim, Germany) was dissolved in DMEM to obtain 25 µg/mL and 75 µg/mL solutions. 25 µg/mL approximates the often-used dose of oxytetracycline in foals of 44 mg/kg calculated based on the following formula as described by Arnoczky et al. (2004): C (µg/mL) = dose (mg/kg)/volume of distribution (L/kg), C being the maximal theoretical concentration^8^. As previously described the volume of distribution for oxytetracycline is 2 L/kg^25^.

Incyclinide (MedChemExpress, Monmouth Junction, USA) was dissolved in a solution of dimethyl sulfoxide (DMSO) in DMEM to obtain 5 µg/mL in 0.25% DMSO and 7.5 µg/mL in 0.375% DMSO solutions.

Ilomastat (MedChemExpress, Monmouth Junction, USA) was dissolved in a solution of DMSO in DMEM to obtain 10 µg/mL in 0.5% DMSO and 20 µg/mL in 1% DMSO solutions.

Aprotinin (MedChemExpress, Monmouth Junction, USA) was dissolved in DMEM to obtain 2 µg/mL and 200 µg/mL solutions.

Pentoxifylline (MedChemExpress, Monmouth Junction, USA) was dissolved in DMEM to obtain 3 µg/mL and 12 µg/mL solutions.

BAPN (MedChemExpress, Monmouth Junction, USA) was dissolved in DMEM to obtain 1000 µg/mL and 3000 µg/mL solutions.

### MTS assay

The viability of cells was assessed through a 3-(4,5-dimethylthiazol-2-yl)-5-(3-carboxymethoxyphenyl)-2-(4-sulfophenyl)-2 H-tetrazolium (MTS) assay (CellTiter 96^®^ AQueous One Solution Cell Proliferation Assay, Promega GmbH, Mannheim, Germany) as previously described^26^. The myofibroblasts were expanded to passage 1 (*N* = 5) or 2 (*N* = 1) and seeded into 96-well microtiter plates at a density of 20’000 cells/well. After 48 h, just before reaching 100% confluence, the plates were incubated for 24 h with the pharmacological compounds in the 2 mentioned concentrations, with DMEM only as positive control or with 10% DMSO as negative control. The highest concentration of DMSO solvent in the pharmacological compound solutions being 1%, this was added as control as well. The MTS was then used in agreement with the manufacturer’s guidelines. The plate was incubated with the MTS for 1 h and the absorbance was measured at 490 nm using a 96-well microtiter plate reader (MRX microplate reader, Dynatech Laboratories, El Paso, US) and expressed in OD-values. The MTS assay was performed in six biological replicates and eight technical replicates for each compound and concentration.

### Crystal violet assay

To evaluate the proliferation capacity of the cells, a modified crystal violet staining assay was performed as previously described^26^. Myofibroblasts were expanded to passage 1 (*N* = 5) or 2 (*N* = 1) and seeded into 96-well microtiter plates at a density of 20’000 cells/well. After 24 h, at 60% confluence, the plates were incubated for 24 h with the pharmacological compounds in the 2 mentioned concentrations. DMEM, DMSO 10% and DMSO 1% were added as control as mentioned above. After discarding the medium-compound mix, the cells were fixed for 20 min using 2% glutaraldehyde (Sigma-Aldrich, Steinheim, Germany) in PBS. After that glutaraldehyde was discarded and 0.1% crystal violet (Roth GmbH, Karlsruhe, Germany) in deionized water was added to each well for 30 min. The plates were then washed using deionized water and allowed to air dry. Once air dried, the dye was solubilized out of the cells by addition of 2% Triton X-100 (Sigma-Aldrich, Steinheim, Germany) in deionized water. Absorbance was measured photometrically at 570 nm using a 96-well microtiter plate reader (MRX microplate reader, Dynatech Laboratories, El Paso, US) and expressed in OD-values. The proliferation assay was performed in six biological replicates and eight technical replicates for each compound and concentration.

### Statistical analysis

Statistical analysis was performed with GraphPad Prism (GraphPad Software, San Diego, CA, USA). Differences between the groups were tested through a Friedman ANOVA test and a Dunn’s test was used to test for pairwise differences between groups. Statistical significance was set at *p* ≤ 0.05. *P* ≤ 0.01 was considered very significant and *p* ≤ 0.001 highly significant.

## Results

### Cell culture

**Fig. 1:**
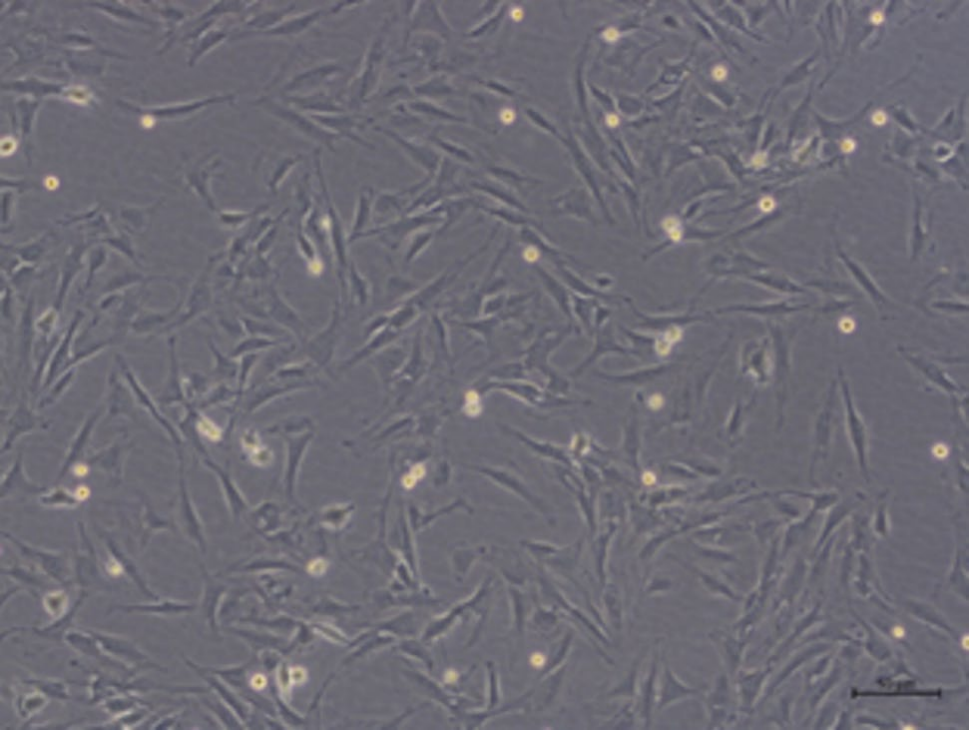
Representative phase contrast micrograph of cultured myofibroblasts from the accessory ligament of the DDFT, scale bar = 50 μm.

The microscopic appearance (Fig. 1) of the cultured cells presented an elongated, spindle-shaped, fibroblast-like morphology, with several membrane protrusions which was consistent with previously described cultured human and equine tendon/ligament myofibroblasts^27,28^. The microscopic appearance did not differ between SDFT and ALDDFT cells.

### Immunohistochemistry

**Fig. 2:**
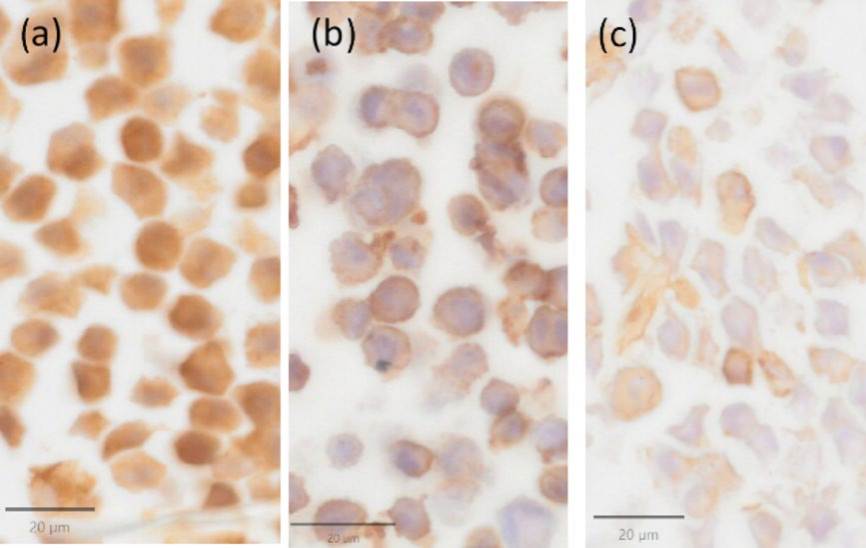
Immunohistochemistry for the detection of collagen type I (**a**), collagen type III (**b**) and alpha-SMA (**c**) in myofibroblasts of the accessory ligament of the DDFT, scale bar = 20 μm.

Immunohistochemistry revealed that collagen type I was present in all constituents of the cell and expressed by 100% of SDFT and ALDDFT cells (Fig. 2a).

Collagen type III was present primarily in the cell membrane and expressed by more than 70% of the cells without difference between SDFT and ALDDFT cells (Fig. 2b).

More than 98% of SDFT cells and between 15% and 60% (median 50%) of the ALDDFT cells expressed α-SMA (Fig. 2c). The marker was present mostly in the cell membrane and present in all constituents of the cell in a single SDFT sample.

The expression profile of collagen types is consistent with tendon/ligament myofibroblasts^27,29^.

### MTS assay

**Fig. 3:**
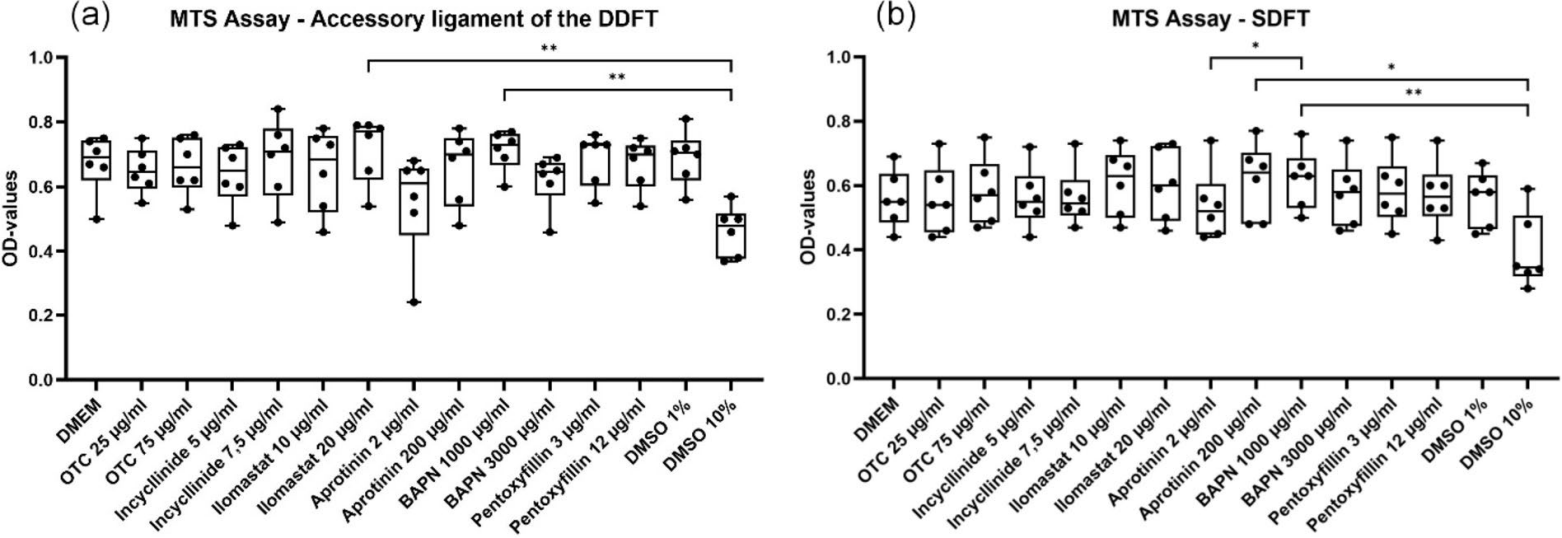
Box plot showing optical density- (OD-)values for myofibroblasts of the accessory ligament of the deep digital flexor tendon (DDFT) (**a**) and superficial digital flexor tendon (SDFT) (**b**) incubated with Dulbecco’s modified eagle medium (DMEM), oxytetracycline (OTC) (25 µg/mL; 75 µg/mL), incyclinide (5 µg/mL; 7.5 µg/mL), ilomastat (10 µg/mL; 20 µg/mL), aprotinin (2 µg/mL; 200 µg/mL), pentoxifylline (3 µg/mL; 12 µg/mL), β-aminopropionitrile fumarate (BAPN) (1000 µg /mL; 3000 µg /mL), dimethyl sulfoxide (DMSO) 1% and DMSO 10% in 3-(4,5-dimethylthiazol-2-yl)-5-(3-carboxymethoxyphenyl)-2-(4-sulfophenyl)-2 H-tetrazolium (MTS) assay; * indicates *p* ≤ 0.05; ** indicates *p* ≤ 0.01; *** indicates *p* ≤ 0.001.

Compared to DMEM, DMSO 1%, and oxytetracycline, no significant differences (*p* > 0.05) were observed for cells from both SDFT and ALDDFT in the MTS assays (Fig. 3a).

In the MTS assay for SDFT cells a significant difference between the OD-values for the cells incubated with aprotinin 2 µg/mL (median 0.52) and those incubated with BAPN 1000 µg/mL (median 0.63; *p* = 0.0262) was observed (Fig. 3b).

ALDDFT and SDFT cells treated with DMSO 10% showed lower median values than those treated with all substances in MTS assays. For ALDDFT cells, this difference was very significant with BAPN 1000 µg/mL (*p* = 0.0077) as well as with ilomastat 20 µg/mL (*p* = 0.0043) and for SDFT cells significant with aprotinin 200 µg/mL (*p* = 0.0189) and very significant with BAPN 1000 µg/mL (*p* = 0.0026) (Fig. 3).

### Crystal violet assay

**Fig. 4:**
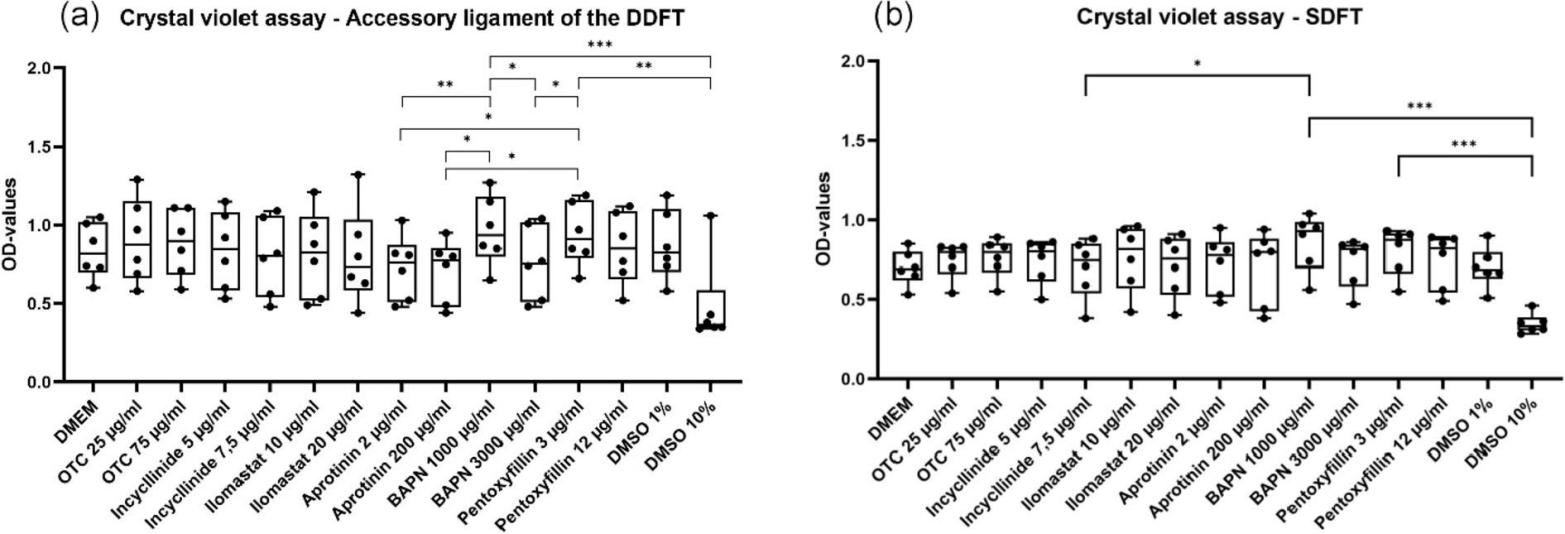
Box plot showing optical density- (OD-)values for myofibroblasts of the accessory ligament of the deep digital flexor tendon (DDFT) (**a**) and superficial digital flexor tendon (SDFT) (**b**) incubated with Dulbecco’s modified eagle medium (DMEM), oxytetracycline (OTC) (25 µg/mL; 75 µg/mL), incyclinide (5 µg/mL; 7.5 µg/mL), ilomastat (10 µg/mL; 20 µg/mL), aprotinin (2 µg/mL; 200 µg/mL), pentoxifylline (3 µg/mL; 12 µg/mL), β-aminopropionitrile fumarate (BAPN) (1000 µg /mL; 3000 µg /mL), dimethyl sulfoxide (DMSO) 1% and DMSO 10% in crystal violet assay; * indicates *p* ≤ 0.05; ** indicates *p* ≤ 0.01; *** indicates *p* ≤ 0.001.

No significant differences (*p* > 0.05) between the OD-values for the cells incubated with the tested substances and those incubated with DMEM, DMSO 1% and oxytetracycline were observed in the crystal violet assays (Fig. 4). That was the case for cells from both SDFT and ALDDFT.

In the crystal violet assay for ALDDFT cells significant differences in OD-values were observed between aprotinin 2 µg/mL (median 0.76) and pentoxifylline 3 µg/mL (median 0.91; *p* = 0.0151), aprotinin 200 µg/mL (median 0.77) and BAPN 1000 µg/mL (median 0.93; *p* = 0.0121), aprotinin 200 µg/mL (median 0.77) and pentoxifylline 3 µg/mL (median 0.91; *p* = 0.0361), BAPN 3000 µg/mL (median 0.75) and BAPN 1000 µg/mL (median 0.93; *p* = 0.0108), as well as between BAPN 3000 µg/mL (median 0.75) and pentoxifylline 3 µg/mL (median 0.91; *p* = 0.0325). The difference was very significant between aprotinin 2 µg/mL (median 0.76) and BAPN 1000 µg/mL (median 0.93; *p* = 0.0048) (Fig. 4a).

In the crystal violet assay for SDFT cells, a significant difference between the OD-values for the cells incubated with incyclinide 7.5 µg/mL (median 0.74) and those incubated with BAPN 1000 µg/mL (median 0.93; *p* = 0.0361) was observed (Fig. 4b).

ALDDFT and SDFT cells treated with DMSO 10% showed lower median values than those treated with all substances in crystal violet assays. For ALDDFT and SDFT cells this difference was respectively very and highly significant with pentoxifylline 3 µg/mL (*p* = 0.0012 for ALDDFT; *p* < 0.0001 for SDFT) and highly significant with BAPN 1000 µg/mL (*p* = 0.0003 for ALDDFT; *p* = 0.0009 for SDFT) (Fig. 4).

## Discussion and conclusion

Oxytetracycline is a useful and widely used adjunctive therapy of flexural limb deformities in foals^4,5^. Its antimicrobial properties, as well as its potential negative side effects, call for the investigation of alternative substances with similar relaxing effects on the muscle-tendon-units of foals and several mechanisms of action, and thereby classes of substances, have been considered in the past. As the inhibition of MMP is currently the favoured hypothesis, as suggested by Arnoczky et al. (2004), four substances with MMP-inhibitor properties (ilomastat, incyclinide, aprotinin and pentoxifylline) were chosen in this study^8^. Moreover, a collagen-cross-linking inhibitor (BAPN) was chosen^13^.

In the performed MTS and crystal violet assays, none of the substances showed a negative impact on the viability and proliferation capacity of the cultured cells in the analyzed concentrations, shown by the absence of statistically significant differences between the tested substances and the control substance DMEM. These properties were similar to oxytetracycline for all tested substances. Due to the intrinsic variable nature of primary cell cultures and the absence of differences between the examined substances and the control substance DMEM, the significant differences found between mentioned test substances cannot be interpreted equivalent to a biologic in vivo result.

Dahlgren et al. (2001) investigated the effect of BAPN on SDFT explants and described an effect of BAPN on collagen synthesis and cell morphology^30^. However, they did not perform any viability or proliferation assays. Moreover, while it has been demonstrated by Arnoczky et al. (2004) that OTC does not affect the viability of cultured myofibroblasts of foals in a live-to-dead assay using ethidium homodimer-1 and calcein AM^8^, to the best of the authors’ knowledge it is the first time that the effect of five alternative substances on juvenile equine myofibroblasts in cell viability and proliferation assays has been tested comparatively. The experiments conducted in this study demonstrate the biocompatibility of all tested substances with cultured juvenile equine myofibroblasts.

In this study, the OD-values for cells treated with DMSO 1% did not differ significantly from those treated with DMEM in MTS and crystal violet assays. These results differ from some previous studies. DMSO has indeed been shown to be toxic for different mammalian cell populations in vitro in a dose-dependent manner, and Singh et al. (2017) showed a negative impact on goat fibroblast cell viability at concentrations higher than 0.5%^31^. Consequently, this threshold does not seem to be applicable to juvenile equine myofibroblasts as a concentration of 1% DMSO seems to be well tolerated and the use of DMSO as solvent in concentrations up to 1% must be deemed safe for juvenile myofibroblasts. However, DMSO, used in a higher concentration of 10% as a negative control, caused a clear negative trend of the OD-values for all substances as expected, reaching statistical significance (*p* < 0.05) in several cases. Further experiments are needed to investigate the lowest toxic concentration of DMSO on this cell type.

The concentration of the test substances can be seen as a limiting factor of this study. The applied concentrations for each substance were chosen according to the published literature. Some substances have been used in in vitro studies using different cell populations: Myers et al. (1998) found that incyclinide in a concentration of 5 µg/mL completely inhibited the contraction of a fibroblast-populated collagen lattice, while Golub et al. (1991) found a 77% in vitro inhibition of collagenase at a concentration of 20 µM (7.427 µg/mL)^32,33^. Ilomastat in concentrations ranging from 10 µM (3.88 µg/mL) to 50 µM (19.4 µg/mL) has been used successfully to inhibit MMPs in the literature^19,34^. In an in vitro experiment on explant SDFT cultures investigating collagen synthesis, Dahlgren et al. (2001) used a 3 mg/mL concentration.

Some other concentrations were adapted from clinical applications: Alves et al. (2001) described the intralesional use of 3 mL of a 0.8 mg/mL BAPN solution in horses for the treatment of tendinopathies^35^. Concentrations of pentoxifylline were adapted from a pharmacokinetic study which found maximal serum concentrations ranging from 2.41 µg/mL to 11.7 µg/mL depending on the way of administration, using a standard dosage of 8.5 mg/kg IV or 10 mg/kg orally in horses^21,36^. Finally, for the concentrations of aprotinin, on the one hand, information from the manufacturer advises concentrations from 0.06 to 2 µg/mL for in vitro experiments, and on the other hand, concentrations ranging from 0.84 mg/mL to 1.753 mg/mL have been described for intralesional administration^20,37^. To reflect the different uses of these substances and the range of concentrations found in the literature, two concentrations were chosen for all test substances. However, the relevant concentration to be administered in the vascular system for the treatment of flexural limb deformities could be outside of the tested range and could lead to different results in MTS and crystal violet assays.

The substances were chosen based on previous reports addressing the most probable mechanisms of action and potential effects on equine tendons and ligaments^8,13^. The selected substances find applications in several medical fields in very different ways. While MMP-inhibitors such as incyclinide are being used in clinical trials in the field of oncology and administered orally, others such as BAPN or aprotinin have been administered intralesionally in equine and human orthopaedics^18,22,35^. Contrary to aprotinin, which has also been safely used systemically to reduce blood loss in human open heart and bypass surgery^38^, BAPN has been shown to cause skeletal pathologies (osteolathyrism) in rats after systemic applications^39^. Aside from the intralesional application of BAPN described in equine tendons, pentoxifylline is the only substance from the panel for which a systemic application has been reported clinically in horses^21^. These considerations highlight the importance of interpreting the current preliminary results with caution from a clinical point of view and the need for further studies to elaborate a safe and efficient way of administering a potential alternative to oxytetracycline.

Collagen type I is the principal component of the extra-cellular matrix of healthy tendons, with collagen type III being also present in healthy tendons, however in a much lower quantity^40^. The expression of both collagen types could be observed in the cultured cells with a predominance of collagen type I. Consequently, as determined by immunohistochemistry, the expression profile of collagens was consistent with tendon/ligament myofibroblasts.

Hartzel et al. (2001) reported that most of the cells found in the ALDDFT and DDFT of foals stained positive for α-SMA, demonstrating that they were myofibroblasts^9^. In the experiments described here, differences between SDFT and ALDDFT cells could be observed, and more variability was observed in ALDDFT cells. The small sample size, i.e. 2 and 3 individuals for SDFT and ALDDFT, respectively, make it difficult to interpret the results but could imply differences in contractile potential. It cannot be excluded that the cell culture, the passaging and the pelleting of the cells altered the expression of α-SMA. A foal’s age at the time of treatment with oxytetracycline significantly influences the potential response to therapy, with younger foals generally responding better^1,5^. However, in one study even up to 3 months old foals showed a decrease in dorsal metacarpophalangeal angle after administration of oxytetracycline^5^. In the current study, no pattern could be observed indicating an influence of the foals’ age on the expression of α-SMA. Further studies with a higher number of individuals are necessary to evaluate potential age-dependent differences in α-SMA expression.

The superficial and the deep digital flexor tendon units, composed of the respective flexor tendon and their accessory ligaments, can be involved in flexural limb deformities^1,2^. The ALDDFT plays an important role in flexural limb deformities as desmotomy of this structure has been shown to be clinically effective in most cases^1,2^. Correspondingly, the effect of oxytetracycline on ALDDFT myofibroblasts has been evaluated in vitro, showing the inhibition of collagen gel contraction by equine myofibroblasts treated with OTC^8^. Cells from the SDFT were additionally included in this study to allow comparison between a tendinous and a ligamentous structure of the flexor tendon unit on the one hand and between a representative structure of the deep and the superficial tendon unit on the other hand. However, testing the substances on cells from the deep digital flexor tendon may provide complementary information.

The characterization of cultured cells through microscopic observation and immunohistochemistry was consistent with tendon/ligament myofibroblasts. However, these methods have limitations as neither of these, nor the chosen markers are entirely specific. An extended panel of markers, including e.g. scleraxis, tenascin-C or tenomodulin, more selective markers such as Eyes absent homolog 2 (EYA2) or G-protein regulated inducer of neurite outgrowth 3 (GPRIN3) recently described or the use of RT-qPCR could have increased the specificity of the characterization^29,41,42^.

In conclusion, this study shows encouraging preliminary results proving the biocompatibility of several MMP-inhibitors and BAPN with juvenile SDFT and ALDDFT myofibroblasts in the analyzed concentrations. Substances were chosen for their chemical properties and potential to influence the contractility of juvenile equine muscle-tendon-units. Consequently, further investigations of the inhibitory capacity of the tested substances on the contraction of juvenile equine muscle-tendon-units are necessary to get further insight into their potential usefulness for the treatment of flexural limb deformities in foals. In a next phase, an in vitro collagen contraction assay will be applied to evaluate the inhibitory capacity of the tested substances on the contraction of a collagen gel seeded with juvenile equine myofibroblasts. Moreover, data on the potential side effects, pharmacodynamics and -kinetics, as well as potential ways of administration of the tested substances in the juvenile equine patient are lacking and these aspects will have to be examined before a credible alternative to oxytetracycline emerges.

## Data Availability

The datasets generated and analysed during the current study are available from the corresponding author on reasonable request.
